# A 5-year retrospective review of post-Mohs reconstruction outcomes for periocular cutaneous malignancies at an academic medical center

**DOI:** 10.3389/fopht.2026.1777608

**Published:** 2026-04-28

**Authors:** Amanda K. Hertel, Aaron Veenis, Rachel Chu, Nathaniel Cameron, Nikki Gill, Irfan Ansari, Maggie Malmberg, Emilee Wells, Geethanjalee Mudunkotuwa, Isuru Ratnayake, Jason A. Sokol

**Affiliations:** 1 Department of Ophthalmology, The University of Kansas School of Medicine, Kansas City, KS, United States; 2 Department of Biostatistics and Data Science, The University of Kansas Medical Center, Kansas City, KS, United States

**Keywords:** post-Mohs reconstruction, skin cancer, periocular malignancies, Mohs surgery, oculoplastics

## Abstract

**Purpose:**

This project examines outcomes of post-Mohs reconstruction for periocular cutaneous malignancies. These results highlight reconstructive surgery challenges and their solutions, a topic with limited peer-reviewed literature.

**Methods:**

A retrospective chart review on post-Mohs reconstruction outcomes for adults seen at a tertiary referral center between August 2018 and March 2023 was performed. Information on demographics, past medical history, and surgical outcomes was collected. Descriptive statistics and correlations where applied were applicable. The Institutional Review Board (IRB) approved this study.

**Results:**

A total of 194 patients met the inclusion criteria (mean age, 67.7 years). Past medical history and skin cancer risk factors were evaluated. The most common periocular malignancy was basal cell carcinoma (70.6%), the location was lower lid (56.7%), and the reconstruction type was Modified Hughes tarsoconjunctival flap (24.7%). There were no intraoperative complications. The most common post-operative complications were healing issues (6.7%) or ectropion (6.2%). Patients reported post-operative symptoms such as eye irritation (23.2%) or pain (20.1%). Most patients had full eyelid function after surgery (93.3%) and acceptable cosmetic appearance (94.8%). Some correlations were found. For example, post-Mohs defect size was associated with variables such as cure rate (p = 0.01834) and general eyelid reconstruction (p = 0.0230). Adjuvant chemotherapy was associated with decreased cure rates (p = 0.0010) and recurrence (p = 0.0174).

**Conclusions:**

This study found associations between health history, periocular cutaneous malignancy features, and post-Mohs reconstruction outcomes. Features such as location, defect size, and staged reconstruction techniques in particular had an association with numerous outcomes.

## 1 Introduction

Skin cancer, specifically basal cell carcinoma, is the most common malignancy affecting the eyelid. Other relatively common periocular cutaneous malignancies include squamous cell carcinoma, sebaceous carcinoma, and melanoma (1). While basal cell carcinoma is the most common periocular cutaneous malignancy worldwide, there is variation among different populations. For example, sebaceous cell carcinoma is much more common in some countries in Asia (2). Periocular cutaneous malignancies make up 5 to 10% of all skin cancers (1). Mohs micrographic surgery (MMS) is the preferred treatment to maximize the cure rate while minimizing damage to surrounding structures (3, 4). This is particularly relevant in periocular cutaneous malignancies, as the eyelid serves to protect the globe and cornea, promote normal tear function, and contribute to cosmesis (4). The margin of resection (MOR) after MMS is typically over 3 times larger in area than the original lesion to ensure adequate excision and is dependent on features like type, size, and location (5). Various methods have also been utilized to try and predict the post-Mohs defect size (6).

**Figure 1:**
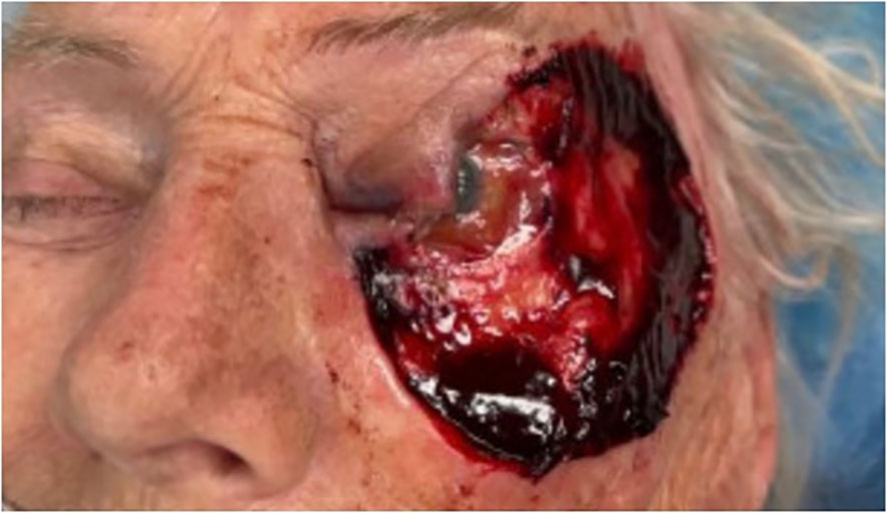
Post-Mohs defect after excision of melanoma involving the left eyelid and lateral canthus. Pre-Reconstruction.

**Figure 2:**
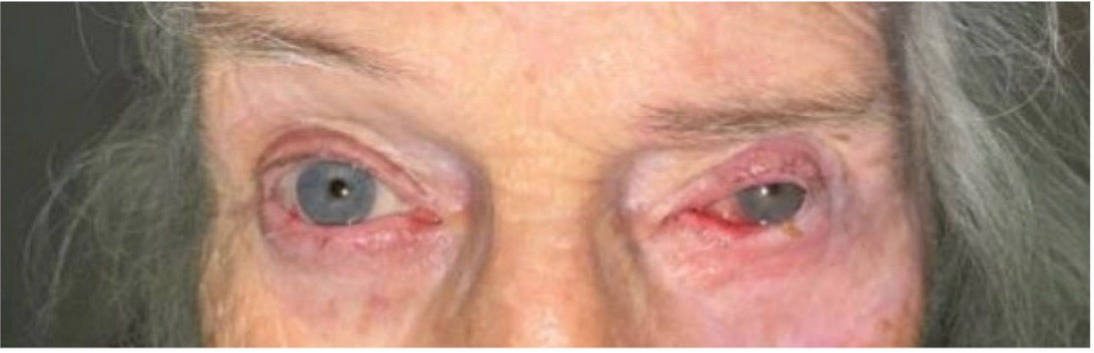
Post-Mohs defect after excision of melanoma involving the left eyelid and lateral canthus. One-year Post-Mohs Reconstruction.

The resulting defect after excision by Mohs requires reconstruction with careful consideration of eyelid anatomy, function, and cosmesis (**Figures 1**, **2**) (3). The method used for reconstruction is dependent upon the defect size and location (7). Direct closure is often preferred; however, this is not always possible depending on size and other features (7). In these cases, more complex closure methods such as Modified Hughes tarsoconjunctival flap or lateral canthotomy Tenzel flap are employed (7). The specific method selected depends upon factors such as the defect location and size as well as factors individual to each patient (8). Treatment and subsequent reconstruction are essential to reduce mortality and morbidity. However, complications (for example, hypertrophic scars, ectropion, or infection) sometimes occur during or after reconstruction (9). Another feared complication is recurrence, estimated to occur in around 0.7 to 5.9% of cases (10). Risk factors for recurrence include things such as larger tumor size or aggressive sub-type (11). Those with more aggressive sub-types also need closer follow-up. However, there is no need to reoperate with small margins in patients with basal cell carcinoma (11). In addition to MMS and reconstruction, adjuvant therapies such as radiation, chemotherapy, and other topical medications may also be indicated (1).

The proposed research intends to examine outcomes of post-Mohs reconstruction for periocular skin cancers treated at an academic medical center. As there is limited peer-reviewed literature on this topic, the results will highlight the challenges of reconstructive surgery and their solutions, ultimately guiding oculoplastic surgeons in achieving optimal results.

## 2 Methods

This study is a retrospective chart review of post-Mohs reconstruction outcomes for periocular cutaneous malignancies. The inclusion criteria for the study included patients over the age of 18 who were seen at the University of Kansas Medical Center (KUMC) between August 2018 and March 2023 and underwent surgery by one oculoplastic surgeon billed under CPT codes 14060, 14061, 14040, 14041, 15771, 15733, 67971, 67973, 67974. Information, including demographics, past medical history, past ocular history, and the details and outcomes of reconstructive surgery, was collected from patient charts. All data was deidentified after collection.

Descriptive statistics were used to summarize the demographic and clinical characteristics of the sample. Outliers were retained in variables such as defect area size, because they represent plausible values and reflect the observed data. Pearson correlations were performed between continuous variables. Linear regression was used in cases of continuous outcome variables. Logistic regression, Chi-squared, and Fisher’s exact tests were used to investigate associations between categorical variables. The statistical analysis was carried out using R statistical software (version 4.4.3). The Institutional Review Board (IRB) has approved this study. This research adhered to the tenets of the Declaration of Helsinki. Written consent was obtained from the patient in **Figures 1**, **2**.

## 3 Results

A total of 194 patients met study inclusion criteria with a mean age of 67.7 years (*SD* = 12.6). There were roughly even numbers of males (53.6%, N = 104) and females (46.4%, N = 90). Most patients were white (94.8%, N = 184) and identified as not Hispanic or Latino (96.9%, N = 188). Past medical history was evaluated with patients having a history of hypertension (HTN; 61.9%, N = 120), hyperlipidemia (HLD; 49.0%, N = 95), Coronary Artery Disease (CAD)/Myocardial Infarction (MI) (25.3%, N = 49), tobacco use history (26.8%, N = 52), and diabetes mellitus (DM; 25.3%, N = 49). Around a quarter of patients had a prior medical history of skin cancer (25.3%, N = 49), with the most common being basal cell carcinoma (N = 17) and squamous cell carcinoma (N = 15). Skin cancer risk factors were evaluated, including excessive sun exposure (13.9%, N = 27), immunosuppression (12.4%, N = 24), fair complexion (6.7%, N = 13), and prior radiation therapy (5.7%, N = 11). Some patients also had a prior history of extraocular surgery (10.3%, N = 20) (**Table 1**).

**Table 1:** Patient demographics, past medical, and past ocular history.


Gender	N (N = 194)	%
Female	90	46.4
Male	104	53.6
Race		
Caucasian	184	94.8
Unknown/Not Reported	8	4.1
Native Hawaiian or Other Pacific Islander	1	0.5
American Indian/Alaska Native	1	0.5
Ethnicity		
Not Hispanic or Latino	188	96.9
Hispanic or Latino	3	1.5
Unknown/Not Reported	3	1.5
Skin cancer history		
History of Skin Cancer	49	25.3
If yes, skin cancer diagnosis (may have more than one diagnosis),		
Basal cell carcinoma	27	13.9
Squamous cell carcinoma	24	12.4
Melanoma	6	3.1
Melanoma in-situ	1	0.5
Unknown	2	1.5
Skin cancer risk factors		
Excessive Sun Exposure	27	13.9
Previous Radiation Therapy	11	5.7
Immunosuppression/Immunocompromised	24	12.4
Fair Complexion/Celtic or Scandinavian Ancestry	13	6.7
No Skin Cancer Risk Factors	121	62.4
Past medical history		
Hypertension (HTN)	120	61.9
Hyperlipidemia (HLD)	95	49.0
Diabetes Mellitus (DM)	49	25.3
Smoking/Tobacco Use	52	26.8
Alcohol Use	33	17.0
Obesity	27	13.9
History of Stroke	12	6.2
Coronary Artery Disease (CAD)/Myocardial Infarction (MI)	49	25.3
Peripheral Artery Disease (PAD)	9	4.6
Chronic Kidney Disease (CKD)	8	4.1
Poor Nutrition	4	2.1
Past ocular history		
History of Extraocular Surgery	20	10.3
Type of extraocular surgery		
Blepharoplasty	3	1.5
Ptosis Repair	0	0
Entropion Repair	0	0
Ectropion Repair	2	1.0
Canthoplasty	3	1.5
Other	10	5.2
Temp Tarsorrhaphy	2	1.0
Muscle Surgery - Unspecified	1	0.5
Lower eyelid wedge resection for trichiasis	1	0.5
Reconstruction of nasal defect with local flap	1	0.5
Dacryocystorhinostomy, crawford tube	1	0.5
Eyelid/Eyelash Surgery - Otherwise Unspecified	1	0.5
Frontal/Transconjunctival orbitotomy with removal of bone for decompression ethmoidectomy	1	0.5
Conjunctivodacryocystorhinostomy	1	0.5

The most common periocular cutaneous malignancy in this study was basal cell carcinoma (70.6%, N = 137), followed by squamous cell carcinoma (19.1%, N = 37), melanoma (7.2%, N = 14), and sebaceous carcinoma (3.1%, N = 6). The most common locations were the right (28.9%, N = 56) and left (27.8%, N = 54) lower lid. A biopsy was performed in most cases (78.4%, N = 152). Few cases were also treated with adjuvant chemotherapy (1.5%, N = 3), with chemotherapy agents being Everidge (1.0%, N = 2) or Capecitabine (0.5%, N = 1). The mean post-resection defect length was 2.64 cm (SD = 2.64 cm), and the mean post-resection defect width was 2.22 cm (SD = 2.84 cm). The mean post-resection defect area was 12.9 cm^2^ (SD = 76.4, range = [0.04, 900]). Some cases did involve the eyelid margin (39.2%, N = 76). The most common reconstruction types were Modified Hughes tarsoconjunctival flap (24.7%, N = 48), lateral canthotomy Tenzel flap (19.1%, N = 37), and direct closure (18.0%, N = 35). Around a quarter of procedures were staged in two or more operations (25.3%, N = 49). There were no intraoperative complications (**Table 2**).

**Table 2:** Periocular cutaneous malignancy Mohs and reconstruction details.


Type of skin cancer	N (N = 194)	%
Basal Cell Carcinoma	137	70.6
Melanoma	14	7.2
Sebaceous Carcinoma	6	3.1
Squamous Cell Carcinoma	37	19.1
Location of skin cancer		
Left Lateral Canthus	5	2.6
Left Lower Lid	54	27.8
Left Medial Canthus	13	6.7
Left Upper Lid	16	8.2
Other/Multiple	18	9.3
Right Lateral Canthus	4	2.1
Right Lower Lid	56	28.9
Right Medial Canthus	22	11.3
Right Upper Lid	6	3.1
Biopsy performed	152	78.4
Adjuvant chemotherapy	3	1.5
Specific chemotherapy agent used,		
Everidge	2	1.0
Capecitabine	1	0.5
Post resection defect area (cm^2^)		
Mean (SD)	12.9 (76.4)	
Median [Min, Max]	3.24 [0.0400, 900]	
Eyelid margin involvement	76	39.2
Type of reconstruction		
Direct Closure	35	18.0
Lateral Canthotomy Tenzel Flap	37	19.1
Cutler Beard	1	0.5
Modified Hughes tarsoconjunctival flap	48	24.7
Free Tarsoconjuctival Flap from Contralateral Lid	11	5.7
Other	61	31.4
Bilobed glabellar flap	13	6.7
Myocutaneous flap	23	11.9
Nasal Sidewall Flap	1	0.5
O-Z Flap	4	2.1
Pedicle Flap	18	9.3
Staged Reconstruction	49	25.3

While most patients had initial follow-up within one month of the procedure, patients were followed for an average of 61 days post-operation (*SD* = 83.99, Median = 18, *IQR* = 81.32, Range = [1,547.5] days). Post-operative complications were evaluated, the most common being healing issues (6.7%, N = 13), cicatricial ectropion (6.2%, N = 12), and infection (4.1%, N = 8). Sixteen post-operative complications occurred in the first month, 20 between months 1 - 4, and 11 after 4 months. Patients also reported post-operative symptoms such as eye irritation (23.2%, N = 45), pain (20.1%, N = 39), tearing (9.8%, N = 19), and dryness (6.7%, N = 13). Most patients had full eyelid function (complete open and closure) after surgery (93.3%, N = 181) and acceptable cosmetic appearance (94.8%, N = 184). A few patients ended up with keloid-type scars (3.6%, N = 7). Some patients also required post-operative revision (6.7%, N = 13), with the most common revision surgery being ectropion repair (4.6%, N = 9). Of the procedures performed, 96.4% (N = 187) were cured, with recurrence happening in 2.6% of patients (N = 5). Metastasis occurred in 1.5% (N = 3) of patients. A few also had an occurrence of a second skin cancer (4.6%, N = 9). (**Supplementary Table 1**).

Associations between various demographics and history with complications were evaluated (**Supplementary Table 2**). Some notable associations include age at diagnosis, which was associated with lagophthalmos (*p* = 0.042), ectropion (*p* = 0.011), and suture granuloma formation (*p* = 0.015). A history of diabetes was associated with wound dehiscence (*p* = 0.012). A history of CAD or prior MI was associated with post-operative infection (*p* = 0.025). PAD history was associated with infection (*p* = 0.001). A history of skin cancer was also associated with a later occurrence of a second cancer in the same patient (*p* = 0.007). A prior history of extraocular surgeries was associated with a greater number of post-operative complications (*p* = 0.043), infection (*p* = 0.021), poor cosmesis (*p* = 0.008), and need for ectropion repair post-operatively (*p* = 0.021). A history of extraocular surgeries was also associated with the cure rate (*p* = 0.001) and recurrence (*p* = 0.004).

Statistically significant mean differences of post-operative complications were observed for direct closure reconstruction (*p* = 0.032), Modified Hughes reconstruction (*p* = 0.028), and staged reconstruction techniques (*p* = 0.003). Ptosis was associated with Free Tarsoconjunctival Flap from Contralateral Lid surgical construction type (*p* = 0.008) as well as staged reconstruction techniques (*p* = 0.026). Post-operative ectropion was associated with eyelid margin involvement (*p* = 0.005), age at reconstruction (*p* = 0.030), Modified Hughes technique (*p* = 0.010), and staged reconstruction technique (*p* = 0.003). Post-operative pain was associated with Modified Hughes (*p* = 0.010). Symptoms of tearing were associated with Modified Hughes (*p* = 0.021) and staged reconstruction techniques (*p* = 0.030). Dryness was associated with eyelid margin involvement (*p* = 0.031) and Modified Hughes (*p* = 0.0001). Eye irritation was associated with Free Tarsoconjunctival Flap from contralateral lid (*p* = 0.019) and staged reconstruction technique (*p* = 0.030). Eyelid function was associated with eyelid margin involvement (*p* = 0.001), Free Tarsoconjunctival Flap from contralateral lid (*p* = 0.004), and staged reconstruction technique (*p* = 0.0036). Need for revision was associated with Modified Hughes (*p* = 0.015) and staged reconstruction (*p* = 0.004). The cure rate was marginally associated with the type of skin cancer (*p* = 0.053) and need for adjuvant chemotherapy (*p* = 0.001). Recurrence was associated with Modified Hughes technique (*p* = 0.023). Metastasis was also associated with type of skin cancer (*p* = 0.009) and need for adjuvant chemotherapy (*p* = 0.007).

Post-Mohs Defect Size was associated with cure rate (*p* = 0.018) and need for general eyelid reconstruction for complications (*p* = 0.023). For example, larger Post-Mohs Defect Size was associated with lower cure rate and more frequent need for revision surgeries. Other notable findings included smoking was not associated with post-operative complications (*p* = 0.603). Post-Mohs defect size was not associated with keloid formation (*p* = 0.802). Adjuvant chemotherapy was associated with decreased cure rates (*p* = 0.001) and recurrence (*p* = 0.017).

## 4 Discussion

The most common periocular cutaneous malignancies in this study were basal cell carcinoma (70.6%), followed by squamous cell carcinoma (19.1%), melanoma (7.2%), and sebaceous carcinoma (3.1%). In the general population, around 80 - 96% of all periocular malignancies are basal cell carcinoma, whereas 5 - 10% are squamous cell carcinoma (9, 10, 12). Therefore, our study sample had a higher rate of squamous cell carcinomas. This could potentially be explained by the fact that patients were seen at a tertiary referral center, where patients may have more comorbidities and immunocompromise (risk factors of squamous cell carcinoma). Consistent with our findings, sebaceous cell carcinoma makes up between 1 - 5.5% of all periocular cutaneous malignancies (1). Interestingly, the rate of melanoma was also much higher than expected, with cutaneous melanoma representing less than 1% of all periocular cutaneous malignancies (1). This higher rate of melanoma could again be due to the patient population, and that melanoma may be more aggressive, thus people seek out earlier resection.

Over half of the patients in this study had skin cancer located on the lower lid (56.7%, N = 110). The location of skin cancer matters as the surgical approach and potential complications are dependent upon location. Both the upper and lower lids may be more commonly associated with complications like eyelid retraction, notching, trichiasis, lid margin mucosal overgrowth, and lacrimal system injury (3). The lower lid may be more commonly associated with complications such as cicatricial ectropion, cicatricial entropion, lateral canthal disruption, and lagophthalmos (3). On the other hand, the upper lid may be associated with complications such as poor blink, ptosis, lateral canthal dystopia, and webbing (3). The necessary MOR is also dependent upon location, with cancers around the lateral canthus needing the largest MOR, and lower eyelids needing the smallest MOR (5). Medial canthus lesions generally require significantly more complex reconstruction due to the anatomy and lacrimal drainage system (5). While many patients had periocular cutaneous malignancy affecting their lower lid, this is still a lower percentage than expected based on prior studies. Studies have shown that around 70% of basal cell carcinomas, 68% of squamous cell carcinomas, and 58% of melanomas affect the lower lid (5). However, sebaceous cell carcinoma typically affects the upper lid in 63% of cases (5). A portion of patients in the study also had eyelid margin involvement (39.2%), which may add to surgical complexity and risk of complications.

The standard of care for periocular cutaneous malignancies is surgical resection, often with Mohs micrographic surgery (MMS). However, for certain high-risk tumors, adjuvant radiation therapy or adjuvant chemotherapy may be recommended. One example of adjuvant chemotherapy is Vismodegib, a small molecule inhibitor of smoothened, used for locally advanced or metastatic basal cell carcinoma (13). Additionally, the antimetabolite agent, Capecitabine, has been suggested to prevent the development of squamous cell carcinoma and other cancerous lesions in those who have received solid organ transplants (14). Only 2.5% of the patients in this study received adjuvant chemotherapy with the agents used being Vismodegib (1.0%, N = 2) and Capecitabine (0.5%, N = 1). Patients treated with adjunctive chemotherapy had a higher rate of metastasis (*p* = 0.007) and decreased cure rates (*p* = 0.001), but decreased recurrence rates (*p* = 0.017), likely reflecting the advanced stage at presentation.

In this study, the mean post-resection defect area was 12.9 cm^2^ (SD = 76.4, [0.04, 900]). Note that with removing the outlier, the mean post-resection defect area was 8.3 cm^2^.Studies have shown that larger lesions may require greater MOR for basal cell and squamous cell periocular malignancies (5). Although the mean postoperative defect areas in that study were much smaller than our average, with the basal cell carcinomas having a mean postoperative defect area of 1.75 cm^2^ and squamous cell carcinomas having a mean postoperative area of 3.02 cm^2^ (5). One explanation of our large post-resection defect area was the presence of an outlier at 900 cm^2^.

Reconstruction of the Mohs resection area requires selecting the correct technique based on lesion characteristics such as size and location, while properly distributing tension and maintaining function and aesthetics (3). As mentioned prior, direct closure is the preferred method if possible. However, other methods must sometimes be used for complex lesions, whether due to size or location (7). In this study, common reconstruction methods included Modified Hughes tarsoconjunctival flap (24.7%), lateral canthotomy Tenzel flap (19.1%), and direct closure (18.0%), with some reconstructions staged in two or more operations (25.3%). The Modified Hughes procedure was likely the most common reconstruction method due to this study being performed at a tertiary academic center with large marginal defects. Ultimately, there are many techniques that can be used for each post-Mohs defect. However, factors such as size and location are often key to determining the method required in order to avoid post-operative complications (8).

While no patients in this study had intraoperative complications, there were numerous post-operative complications reported, such as healing issues (6.7%), ectropion (6.2%), and infection (4.1%). Patients also reported post-operative symptoms such as eye irritation (23.2%), pain (20.1%), tearing (9.8%), and dryness (6.7%). One study on Mohs reconstruction outcomes found that complications happened in 23% of cases with common ones being epiphora (7%), hypertrophic scar (7%), followed by ectropion (4%), edema (3%), infection (3%), exposure keratopathy (3%), lagophthalmos (1%), ptosis (1%), and loss of visual acuity (1%) (9). They also reported that 19% of these complications required additional surgery (9), a number significantly more than the 6.7% requiring post-operative revision in our study. Many of these complications occurred in the first 4 months (76.6%, N = 36 out of 47 complications), stressing the importance of early follow-up intervals. It is also noted that in this study, the majority had full eyelid function (93.3%) and acceptable cosmetic appearance (94.8%).

At the time data was collected, 96.4% were cured, with recurrence in only 2.6% of patients. A subset also had metastasis (1.5%) or a second skin cancer diagnosed (4.6%) within the study follow-up period. Our findings are consistent with other papers on periocular cutaneous malignancies, finding recurrence rates between 0.7 - 5.9% (9, 10). Generally, the five-year cure rate is 99% for basal cell carcinoma and 98.1% for squamous cell carcinoma (15). While our cure rate is slightly lower, we also had a larger portion of patients with melanoma, which could explain this difference.

Post-Mohs Defect Size was associated with cure rate (*p* = 0.018) and general eyelid reconstruction (*p* = 0.023). Ultimately, a larger defect size was associated with decreased cure rate. This is likely because larger defects may be associated with more aggressive cancers (5, 6). Additionally, larger post-Mohs defects may require additional and more complex reconstructions (5), hence requiring additional revisions.

Location of the cancer was associated with post-op tearing (*p* = 0.038) and need for ectropion repair (*p* = 0.035). Cancers on the left medial canthus (N = 1) and left upper lid (N = 2) were associated with tearing. Ectropion repair was associated with cancer along the left lateral canthus (N = 1), left medial canthus (N = 1), and right lower lid (N = 7). Having eyelid margin involvement was also associated with dryness (*p* = 0.031), eyelid function (*p* = 0.001), and ectropion formation (*p* = 0.005). As these areas require careful excision due to important nearby structures, it is not surprising that some patients may experience post-operative symptoms. Studies have also reported that lesions at the medial canthus need more complex reconstruction procedures (5). This is likely also true for lesions around the eyelid margin.

In this study, smoking was not associated with post-operative complications (*p* = 0.603). While this result was initially unexpected, other studies have similarly found that smoking is not a statistically significant risk factor for post-operative complications after post-Mohs reconstruction (9).

Our analysis also found that some reconstruction techniques had more complications than others. For example, there was a greater number of post-operative complications for direct closure reconstruction (*p* = 0.032), Modified Hughes reconstruction (*p* = 0.028), and staged reconstruction techniques (*p* = 0.003). Past studies have also evaluated this and found that full-thickness skin grafts and lid grafts had higher rates of complications when controlling for defect size (9).

A strength of this study is the large sample size and data collection over multiple follow-up clinic visits. However, some limitations include the retrospective design taking place at a single academic center (with a single oculoplastic surgeon). Additionally, there can be bias in the findings reported within each note. For example, what is considered ‘acceptable cosmetic appearance’, may be different for each patient and not always be brought up by the patient during the visit. Future studies could explore these outcomes at multiple academic medical centers.

## 5 Conclusion

This study found many associations between health history and periocular cutaneous malignancy features and post-Mohs reconstruction outcomes. Features such as malignancy location, post-Mohs defect size, and staged reconstruction techniques in particular had an association with numerous outcomes. By better understanding how such variables impact post-Mohs reconstruction outcomes, we hope that oculoplastic surgeons can better predict and account for these results.

## Data Availability

The original contributions presented in the study are included in the article/**Supplementary Material**. Further inquiries can be directed to the corresponding author.
