# Supplementary material for: A 5-year retrospective review of post-Mohs reconstruction outcomes for periocular cutaneous malignancies at an academic medical center
Source: Front Ophthalmol (Lausanne). 2026 Apr 28;6:1777608. doi: 10.3389/fopht.2026.1777608 (PMC13160680; doi:10.3389/fopht.2026.1777608)
Supplement: Supplementary file 1 [file Table1.docx]

Supplementary Material

# Supplementary Tables

**Supplementary Table 1.** Periocular cutaneous malignancy reconstruction outcomes.

|  | Overall (N=194) |  |
| --- | --- | --- |
| **Number of post-op period complications** |  |  |
| Mean (SD) | 0.289 (0.619) |  |
| Median [Min, Max] | 0 [0, 4.00] |  |
| **Type of post-op period complication** | N | % |
| Healing issues. | 13 | 6.7 |
| Ectropion. | 12 | 6.2 |
| Infection. | 8 | 4.1 |
| Bleeding | 3 | 1.5 |
| Epiphora Secondary to Punctal Malposition. | 2 | 1.0 |
| Lagophthalmos. | 2 | 1.0 |
| Corneal surface problems. | 1 | 0.5 |
| Lower Eyelid Retraction. | 1 | 0.5 |
| Corneal Abrasion. | 0 | 0 |
| Diplopia. | 0 | 0 |
| Entropion. | 0 | 0 |
| Floppy Eyelid Syndrome. | 0 | 0 |
| Lid Notching or Lid Margin Irregularity | 0 | 0 |
| Other (i.e. necrosis, keloid, edema, erythema) | 7 | 3.6 |
| **Timing of post-op period complication** |  |  |
| 0 <= weeks < 4 | 16 |  |
| 1 <= months < 2 | 9 |  |
| 2 <= months < 3 | 7 |  |
| 3 <= months < 4 | 4 |  |
| 4+ months | 11 |  |
| **Post-op period patient reported symptoms** |  |  |
| Pain | 39 | 20.1 |
| Tearing | 19 | 9.8 |
| Dryness | 13 | 6.7 |
| Eye irritation | 45 | 23.2 |
| Eyelid Drooping | 2 | 1.0 |
| Incomplete Eyelid Closure | 4 | 2.1 |
| Poor cosmesis. | 3 | 1.5 |
| Other | 6 | 3.1 |
| Decreased sensation/numbness | 2 | 1.0 |
| Heavy sensation | 1 | 0.5 |
| Itching | 2 | 1.0 |
| Itching, decreased sensation/numbness | 1 | 0.5 |
| **Eyelid Function** |  |  |
| Can fully close | 181 | 93.3 |
| Can partially close | 10 | 5.2 |
| Cannot close | 1 | 0.5 |
| Missing | 2 | 1.0 |
| **Cosmesis** |  |  |
| Acceptable appearance | 184 | 94.8 |
| Unacceptable appearance | 6 | 3.1 |
| Missing | 4 | 2.1 |
| **Scar type - keloid** | 7 | 3.6 |
| **Need for corneal neurotization** | 0 | 0.0 |
| **Need for revision** | 13 | 6.7 |
| **Need for additional surgeries for complications** |  |  |
| Cryotherapy | 0 | 0 |
| Ectropion repair | 9 | 4.6 |
| Entropion repair | 0 | 0 |
| Excisional Biopsy | 2 | 1.0 |
| Full Thickness Wedge Resection and Repair | 2 | 1.0 |
| General Eyelid Reconstruction. | 3 | 1.5 |
| Incisional Biopsy | 0 | 0 |
| Lateral Canthoplasty | 2 | 1.0 |
| Mohs Micrographic Surgery. | 0 | 0 |
| Orbital Exeneternation | 2 | 1.0 |
| Ptosis repair. | 3 | 1.5 |
| Radiation Therapy | 0 | 0 |
| Systemic Chemotherapy | 0 | 0 |
| Other | 6 | 3.1 |
| Anterior Orbitotomy | 1 | 0.5 |
| Kenolog injection and wound dehiscence repair | 1 | 0.5 |
| Lid retraction repair | 1 | 0.5 |
| Median Tarsorrhaphy | 1 | 0.5 |
| Mucous membrane excision | 1 | 0.5 |
| Wound dehiscence repair | 1 | 0.5 |
| **Cured** |  |  |
| Yes | 187 | 96.4 |
| No | 7 | 3.6 |
| **Recurrence** |  |  |
| No | 189 | 97.4 |
| Yes | 5 | 2.6 |
| **Timing of recurrence** |  |  |
| Less than one-year post-op | 3 | 1.5 |
| Between one and two-years post-op 8 months post op | 2 | 1.0 |
| **Metastasis** |  |  |
| No | 191 | 98.5 |
| Yes | 3 | 1.5 |
| **Patient deceased** |  |  |
| No | 184 | 94.8 |
| Yes | 10 | 5.2 |
| **Second cancer in same patient** |  |  |
| No | 185 | 95.4 |
| Yes | 9 | 4.6 |
| **Specify second cancer** |  |  |
| Basal cell carcinoma | 3 | 1.5 |
| Melanoma | 1 | 0.5 |
| Squamous cell carcinoma | 4 | 2.1 |

**Supplementary Table 2.** Associations between demographics, history, and outcome variables found using linear regression, logistic regression, Chi squared test, or Fisher’s exact test.

| **Outcome variable** | **Demographic variable** | ***p*-value** |
| --- | --- | --- |
| Post-op complication – Lagophthalmos | Age at diagnosis | 0.042 |
| Post-op complication – Ectropion | Age at diagnosis | 0.011 |
| Post-op complication – Suture Granuloma | Age at diagnosis | 0.015 |
| **Outcome variable** | **Past medical history variable** | ***p*-value** |
| Post-op complication - Wound dehiscence | History of Diabetes | 0.012 |
| Post-op complication - Infection | History of coronary artery disease/prior MI | 0.025 |
| Post-op complication - Infection | History of Peripheral Artery Disease | 0.001 |
| Post-op symptoms - Pain | Skin cancer risk factor - Immunosuppression or Immunocompromised | 0.028 |
| Post-op symptoms - Other | History of Prior stroke | 0.018 |
| Need for additional surgeries - Other | History of HLD | 0.013 |
| Need for additional surgeries - Other | History of Alcohol use | 0.008 |
| Need for additional surgeries - Other | History of coronary artery disease/prior MI | 0.037 |
| Recurrence | History of Diabetes | 0.024 |
| Patient Deceased | Skin cancer risk factors – Sun exposure | 0.009 |
| Patient Deceased | History of Obesity | 0.035 |
| Patient Deceased | History of Poor nutrition | 0.014 |
| Second cancer in same patient | History of skin cancer | 0.007 |
| **Outcome variable** | **Past ocular history variable** | ***p*-value** |
| Number of post-op complications | History of Extra Ocular Surgeries | 0.043 |
| Post-op complication - Infection | History of Extra Ocular Surgeries | 0.021 |
| Post-op symptoms– Poor cosmesis | History of Extra Ocular Surgeries | 0.029 |
| Post-op symptoms – Poor cosmesis | Extra ocular surgery - Other | 0.031 |
| Cosmesis | History of Extra Ocular Surgeries | 0.008 |
| Cosmesis | Extra ocular surgery - Other | 0.001 |
| Need for revision | Extra ocular surgery - Other | 0.031 |
| Need for additional surgeries – Ectropion repair | History of Extra Ocular Surgeries | 0.021 |
| Need for additional surgeries – Orbital Exeneternation | Extra ocular surgery - Canthoplasty | 0.031 |
| Need for additional surgeries – General eyelid reconstruction | Extra ocular surgery - Canthoplasty | 0.046 |
| Cure rate | History of Extra Ocular Surgeries | 0.001 |
| Cure rate | Extra ocular surgery - Canthoplasty | 0.003 |
| Recurrence | History of Extra Ocular Surgeries | 0.004 |
| Recurrence | Extra ocular surgery - Canthoplasty | 0.002 |
| **Outcome variable** | **Eyelid cancer summary variable** | ***p*-value** |
| No. of post-op complications | Type of surgical reconstruction performed – Direct Closure. | 0.032 |
| No. of post-op complications | Surgical reconstruction performed – Modified Hughes | 0.028 |
| No. of post-op complications | Reconstruction technique - staged | 0.003 |
| Post-op complication – Tearing | Location of the cancer | 0.038 |
| Post-op complication – Tearing | Adjuvant Chemotherapy | 0.007 |
| Post-op complication – Ptosis | Type of surgical reconstruction performed – Free Tarsoconjuctival Flap from Contralateral Lid | 0.026 |
| Post-op complication – Ptosis | Reconstruction technique - staged | 0.026 |
| Post-op complication – Lagophthalmos | Age at reconstruction | 0.038 |
| Post-op complication – Ectropion | Eyelid margin involvement | 0.001 |
| Post-op complication – Ectropion | Age at reconstruction | 0.030 |
| Post-op complication – Ectropion | Surgical reconstruction performed – Modified Hughes | 0.010 |
| Post-op complication – Ectropion | Reconstruction technique - staged | 0.003 |
| Post-op complication – Other | Age at reconstruction | 0.038 |
| Post-op complication – Other | Type of surgical reconstruction performed – Free Tarsoconjuctival Flap from Contralateral Lid | 0.053 |
| Post-op symptoms – Pain | Surgical reconstruction performed – Modified Hughes | 0.010 |
| Post-op symptoms – Pain | Complication DURING reconstruction surgery | 0.033 |
| Post-op symptoms – Tearing | Surgical reconstruction performed – Modified Hughes | 0.021 |
| Post-op symptoms – Tearing | Reconstruction technique - staged | 0.030 |
| Post-op symptoms – Dryness | Eyelid margin involvement | 0.031 |
| Post-op symptoms – Dryness | Surgical reconstruction performed – Modified Hughes | 0.001 |
| Post-op symptoms – Eye irritation | Type of surgical reconstruction performed – Free Tarsoconjuctival Flap from Contralateral Lid | 0.019 |
| Post-op symptoms – Eye irritation | Reconstruction technique - staged | 0.030 |
| Eyelid function | Eyelid margin involvement | 0.001 |
| Eyelid function | Type of surgical reconstruction performed – Free Tarsoconjuctival Flap from Contralateral Lid | 0.004 |
| Eyelid function | Reconstruction technique - staged | 0.004 |
| Need for revision | Surgical reconstruction performed – Modified Hughes | 0.015 |
| Need for revision | Reconstruction technique - staged | 0.004 |
| Need for additional surgeries– Ptosis repair | Type of surgical reconstruction performed – Free Tarsoconjuctival Flap from Contralateral Lid | 0.009 |
| Need for additional surgeries– Ptosis repair | Reconstruction technique - staged | 0.017 |
| Need for additional surgeries – Ectropion repair | Type of skin cancer | 0.054 |
| Need for additional surgeries – Ectropion repair | Location of skin cancer | 0.035 |
| Need for additional surgeries – Ectropion repair | Eyelid margin involvement | 0.030 |
| Need for additional surgeries – Ectropion repair | Surgical reconstruction performed – Modified Hughes | 0.043 |
| Need for additional surgeries – Ectropion repair | Reconstruction technique - staged | 0.052 |
| Need for additional surgeries – Orbital Exeneternation | Adjuvant Chemotherapy | 0.031 |
| Need for additional surgeries – General Eyelid Reconstruction | Type of skin cancer | 0.046 |
| Need for additional surgeries – General Eyelid Reconstruction | Surgical reconstruction performed – Modified Hughes | 0.014 |
| Need for additional surgeries – Other | Location of skin cancer | 0.041 |
| Cure rate | Type of skin cancer | 0.053 |
| Cure rate | Adjuvant Chemotherapy | 0.001 |
| Recurrence | Surgical reconstruction performed – Modified Hughes | 0.023 |
| Metastasis | Type of skin cancer | 0.009 |
| Metastasis | Adjuvant Chemotherapy | 0.007 |
| Patient Deceased | Type of skin cancer | 0.001 |
| Patient Deceased | Surgical reconstruction performed – Modified Hughes | 0.016 |
| Patient Deceased | Reconstruction technique - staged | 0.020 |
| Second cancer in same patient | Type of skin cancer | 0.032 |
| Cure Rate | Post-Mohs Defect Size | 0.018 |
| Need for additional surgeries for complications – General Eyelid Reconstruction | Post-Mohs Defect Size | 0.023 |
